# Radiation Therapy for Chemotherapy Refractory Gingival Myeloid Sarcoma

**DOI:** 10.3389/fonc.2021.671514

**Published:** 2021-05-10

**Authors:** Daniel Y. Lee, Jonathan Baron, Christopher M. Wright, John P. Plastaras, Alexander E. Perl, Ima Paydar

**Affiliations:** ^1^ University of Pennsylvania School of Medicine, Philadelphia, PA, United States; ^2^ Department of Radiation Oncology, University of Pennsylvania, Philadelphia, PA, United States; ^3^ Department of Medicine, Division of Hematology-Oncology, University of Pennsylvania, Philadelphia, PA, United States

**Keywords:** gingival, myeloid sarcoma, chloroma, radiation, acute myeloid leukemia

## Abstract

Gingival myeloid sarcoma (MS) refractory to induction chemotherapy is a rare clinical entity and can be treated with palliative radiation therapy (RT). However, there are few previously published reports of RT approaches for the treatment of gingival MS. We present a single institution retrospective observational study of adult patients treated with palliative RT for chemotherapy refractory gingival MS. A total of six patients diagnosed with gingival MS in the setting of relapsed or refractory acute myeloid leukemia treated with palliative RT were identified, with a median age of 66 (range 52–77). Patients were treated with radiation doses ranging from 5 to 20 Gy in 2–10 fractions. Two patients had adequate follow-up time to assess treatment response. One patient who was simulated with PET/CT experienced a local complete response, while the other patient required retreatment 2 months after initial treatment and experienced an eventual local partial response. Three patients experienced radiation mucositis, with one patient experiencing grade 5 toxicity attributed to concomitant treatment with the radiosensitizer hydroxyurea. We believe that this study can provide a practical reference point for other clinicians given the rarity of gingival MS requiring palliative radiation therapy as a clinical entity.

## Introduction

Myeloid sarcoma (MS), also known as chloroma or granulocytic sarcoma, is an uncommon extramedullary tumor comprised of immature myeloid precursor cells. It affects about 3–8% of patients with acute myeloid leukemia (AML) (1, 2). However, MS can also occur with other myelodysplastic syndromes, *de novo* as an isolated finding, or as an initial manifestation of AML prior to bone marrow involvement (3). The presence of MS is often associated with a poor prognosis (4).

MS most commonly affects the lymph nodes, soft tissues, gastrointestinal tract, bone, orbits, and skin—however, essentially any extramedullary site in the body may be affected (1). Gingival hyperplasia from leukemic infiltration is considered a type of MS that can significantly affect quality of life for patients. It is classically associated with acute myelomonocytic leukemia and acute monocytic leukemia, as is MS in general (5–7). Manifestations include pain, bleeding, ulceration, infection, difficulty with speech, and difficulty with maintaining oral intake. While gingival leukemic infiltration and myeloid sarcoma in general can resolve with induction chemotherapy, low dose radiotherapy (RT) may play a palliative role for persistent symptoms refractory to chemotherapy (3, 8). However, the efficacy of RT for chemotherapy refractory gingival MS and optimal treatment regimens that minimize radiation mucositis have not been well studied given the rarity of the entity.

We conducted a single institution retrospective analysis of six patients treated with palliative intent radiation for chemotherapy refractory gingival MS as a manifestation of AML. We hope this may provide a practical reference point for other radiation and medical oncologists to assist in the management of gingival MS in advanced AML patients or for patients unable to tolerate intensive therapy who require palliation.

## Methods

A retrospective observational single institution analysis was performed for adult patients treated with palliative RT for gingival MS (either clinically diagnosed or biopsy proven) associated with AML at the University of Pennsylvania from 1/1/2010 to 8/1/2020. This study was approved by the University of Pennsylvania Institutional Review Board.

Electronic medical records were reviewed for patients who met inclusion criteria. Recorded patient specific variables included sex, race, age at treatment, and date of last follow-up or death. Disease specific variables included date of initial cancer diagnosis, date of initial MS diagnosis, symptoms, location within oral cavity, and biopsy details if relevant. Treatment specific variables included systemic therapy prior to MS diagnosis, hematopoietic stem cell transplant (HCT) prior to myeloid sarcoma diagnosis, concurrent systemic therapy during RT, number of RT fractions, RT total dose, and RT retreatment parameters when relevant. Toxicities were retrospectively recorded according to the Common Terminology Criteria for Adverse Events v5.0.

The primary outcome was response to palliative radiation. Local response to radiation was graded as local complete response (CR), local partial response (PR), stable disease, or progressive disease. Though response to anti-leukemia therapy is generally graded systemically *via* the revised criteria set forth by the International Working Group (9), we graded local treatment response to the lesion of interest using the Lugano criteria when appropriate (10). Local CR was considered to be a complete metabolic response on positron emission tomography-computed tomography (PET/CT, when available) or a complete clinical response on physical examination or CT. Local PR was recorded with a decrease in metabolic activity on PET/CT or with a noticeable clinically significant improvement on physical exam or CT. Stable or progressive disease was noted with no improvement or worsening disease, respectively.

Overall survival was calculated from the date of the first radiotherapy treatment to the date of last follow up or death.

## Results

A total of six patients diagnosed with gingival MS and subsequently treated with palliative RT were identified. Patients were either diagnosed clinically (three) or by biopsy (two by direct biopsy, one with biopsy to an adjacent lip lesion found to be leukemia cutis, **Table 1**). The median age was 66 years (range 52–77). All patients were previously diagnosed with relapsed/refractory AML and treated with multiple (three to eight) previous lines of systemic therapy. Four of six patients were treated previously with allogeneic HCT.

**Table 1:** Demographics, Clinical Characteristics, Treatment Parameters, and Outcomes.

	Patient 1	Patient 2	Patient 3	Patient 4	Patient 5	Patient 6
Age (At RT)	71	63	68	52	65	77
Sex	Female	Female	Female	Male	Female	Female
Race	White	White	White	White	White	Not Specified
**Disease Parameters**						
AML subtype	Myeloblastic	Myeloblastic	Monocytic	Myelomonocytic	Myelomonocytic	Unknown
MS location	Gingiva	Gingiva/Hard Palate	Gingiva	Gingiva/Lips/Hard Palate	Gingiva	Hard Palate/Sinus
MS symptoms	Pain, hyperplasia/mass	Pain, hyperplasia, bleeding	Hyperplasia, bleeding	Pain, ulcers	Pain, hyperplasia	Pain
MS Biopsied	Yes	No	No	Yes^†^	No	Yes
Other Extramedullary Disease	None known	Liver	None known	None known	Skin	None known
Systemic treatment during RT	None	Subcutaneous cytarabine	Hydroxyurea	Sorafenib	None	Hydroxyurea
**Treatments Before RT**						
Systemic Treatment Lines (No.)	7	8	3	7	3	4
Cytotoxic Treatments Lines (No.)	3	7	3	4	3	4
Prior Bone Marrow Transplant	Yes	Yes	No	Yes	Yes	No
**RT Parameters**						
Total Dose [fractions] (cGy)	2,000 [10]	1,200 [6]	1,200 [6]	2,000 [10]	500 [2]	2,000 [10]
Re-treatment Dose [fractions] (cGy)	NA	1,000 [5]	NA	NA	NA	NA
Modality	Rapid Arc IMRT	Opposed Laterals	Opposed Laterals	Opposed Laterals	Opposed Laterals	3D Conformal (3-field)
Highest Acute Toxicity Grade	Grade 1 (fatigue)	Grade 3 (mucositis)	None	Grade 2 (mucositis)	None	Grade 5 (mucositis)
**Outcomes**						
In field Clinical Response	CR	PR	NA	NA	NA	NA
Time from treatment to last follow-up or death (Days)	1428	170	23	4	8	18
Survival Status	Alive	Deceased	Deceased	Deceased	Deceased	Deceased

RT, radiotherapy; AML, acute myeloid leukemia; MS, myeloid sarcoma; IMRT, intensity modulated radiation therapy; CR, complete response; PR, partial response; NA, not applicable.

^†^Adjacent lip lesion biopsied as leukemia cutis.

The total RT dose patients were treated with ranged from 5 to 20 Gy in 2–10 fractions (**Table 1**). No patients had prior in-field RT treatment. Four patients experienced treatment toxicity including grade 1 fatigue and grades 2, 3, and 5 mucositis. The patient who experienced grade 5 toxicity received 20 Gy in 10 fractions and developed severe mucositis attributed to concomitant treatment with the radiosensitizer hydroxyurea that resulted in hospitalization, multiple aspirations requiring intubation, and acute respiratory distress syndrome ultimately leading to death. Another patient with FLT3 positive disease who experienced grade 2 mucositis was concomitantly treated with the radiosensitizer sorafenib. Four patients had limited follow-up after treatment due to transfer to hospice care or death that precluded long-term assessment of treatment response (days from last RT to death ranged from 4 to 23 days) while two patients had treatment response recorded. Overall, the median survival from RT is 20.5 days (range 4 days–3.9 years).

**Figure 1:**
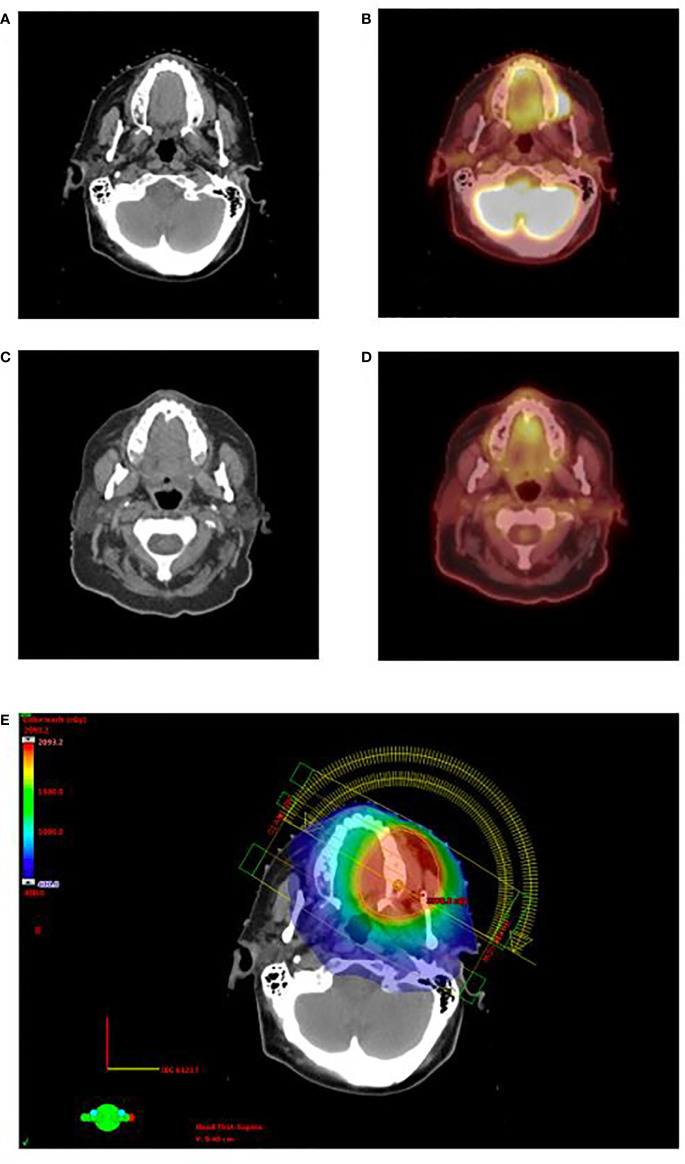
Clinical appearance of gingival MS in the left superior alveolar ridge for patient 1 on pretreatment CT **(A)** and PET/CT **(B)**. The patient demonstrated complete response on CT **(C)** and PET/CT **(D)** 3 months after the completion of RT. Rapid arc IMRT treatment plan is shown in **(E)**. CT, computed tomography; PET/CT, positron emission tomography/computed tomography; RT, radiotherapy; IMRT, Intensity modulated radiation therapy.

Two patients had adequate follow-up time to assess treatment response. One patient was a 71-year-old female with a history of relapsed/refractory AML who presented with biopsy confirmed MS of the left upper buccal gingiva. Her complete blood count at the time suggested continued bone marrow remission with near normal blood counts. She was simulated with positron emission tomography/computed tomography (PET/CT) which demonstrated a markedly hypermetabolic mass in the left superior alveolar ridge consistent with her known MS, and she was treated with palliative intent volumetric modulated arc therapy to a total dose of 20 Gy in 10 fractions (**Figure 1**). She experienced a complete metabolic response on PET/CT 3 months post-RT with no subsequent local relapse, and she experienced no significant side effects. However, she did experience a third systemic relapse of AML in the following months for which she resumed systemic therapy.

**Figure 2:**
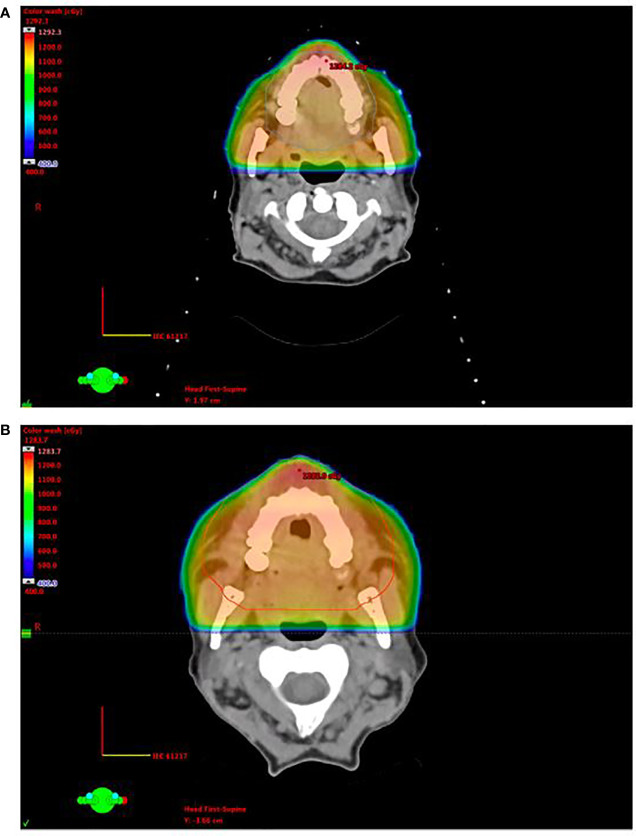
Opposed lateral treatment plans for patient 2, who experienced generalized gingival and hard palate leukemic infiltration, on initial treatment **(A)** and re-treatment **(B)**.

A second patient was a 63-year-old female with active relapsed/refractory AML who presented with diffuse gingival ulcers, bleeding, and hyperplasia while enrolled in a clinical trial where she was receiving eltrombopag and hydroxyurea. She was clinically diagnosed with gingival MS and was treated with palliative intent opposed lateral fields to a total dose of 12 Gy in six fractions with concomitant subcutaneous cytarabine (**Figure 2A**). She experienced grade 3 mucositis that was managed with oxycodone and resolved 1 week after RT completion. She was found to have progression of gingival enlargement 2 months after completion of RT, and she was treated with a second course of palliative intent RT *via* opposed lateral fields to a dose of 10 Gy in five fractions, opting out of her sixth fraction due to a painful lateral tongue ulcer (**Figure 2B**). Follow-up 1 month later demonstrated a local partial response to RT.

## Discussion

MS is a rare extramedullary mass of myeloid precursor cells (3). While MS does not solely occur in the setting of relapsed AML, this was the case for all patients in our study treated with palliative RT. The diagnosis of MS is aided by a history of AML such as for these patients; however, for patients without AML, MS can often be misdiagnosed as a lymphoma or a different entity (3).

We present, to our knowledge, the first analysis of patients treated with RT for gingival MS. There is currently no consensus on the optimal RT treatment schedule for gingival MS given its rarity. In general, RT is indicated in MS for residual disease after chemotherapy, symptomatic relief, isolated MS at presentation, or recurrence after HCT (3).

While low dose RT of 20–24 Gy is generally recommended for MS according to guidelines by the International Lymphoma Radiation Oncology Group (ILROG), even this dose must be weighed against the risk of significant mucositis particularly in the palliative setting (3, 11). However, lower doses such as 6 Gy for gingival MS have been previously reported to result in local failure (8, 11). The patients in this study were treated to doses of 5–20 Gy. While the size of this study is small, the fact that one patient experienced local failure after 12 Gy while another had a complete metabolic response after 20 Gy with no signs of mucositis may suggest 20 Gy in 10 fractions could be a reasonable schedule for further study in gingival MS when local control is important. However, our data also suggest that doses in the 20–24 Gy range may increase toxicity, which may be undesirable given the limited survival. For example, a patient who developed severe mucositis was also treated to 20 Gy in 10 fractions while taking hydroxyurea; this could represent radiosensitization to systemic agents or inflammation related to tumor lysis. Thus, caution is advised when using doses in this range, especially when using concurrent systemic therapy and when survival is anticipated to be short. Ultimately, the desire for local control must be balanced against the risk of toxicity for each patient individually.

It has been demonstrated that PET/CT is a more effective tool than CT alone for detecting MS and has thus been previously suggested to be effective for both RT planning and response evaluation (11–13). One patient in this study was evaluated with PET/CT before and after RT treatment, and the images we present re-demonstrate the advantages of PET/CT over CT alone. This case is also an example of the usefulness of PET/CT for gingival MS given that metabolic activity after treatment corresponded well with treatment response and durability. Additionally, PET/CT is particularly important as it can detect additional sites of MS that were not clinically apparent—60% of MS patients in a study by Stölzel et al. were found to have additional sites of extramedullary disease after evaluation by PET/CT (13). Nevertheless, treatment of subclinical sites may increase morbidity without clinical benefit.

With a median survival of 20.5 days after RT, the patients in our study had poor prognoses. Also, MS after HCT is relatively rare and is a sign of poor prognosis as was the case for the patients in our study (14, 15). There have been no randomized prospective trials to address treatments for extramedullary leukemia. Thus, the role of RT for MS remains to be further studied.

The limitations of this study include its small size in addition to its retrospective design. While it is difficult to draw conclusions from a small cohort, these cases are valuable to other clinicians given the rarity of gingival MS requiring palliative RT as a clinical entity.

## Conclusion

This study is the first, to our knowledge, to report different RT treatment approaches for gingival MS. Patients demonstrated clinical response to RT for gingival MS, and 20–24 Gy in 10–12 fractions may represent a reasonable dose for future study in concordance with ILROG guidelines, although the risk of life-threatening mucositis must be monitored particularly while being treated with radiosensitizing systemic agents. Lastly, PET/CT is a useful imaging modality for RT planning as well as evaluation of treatment response in gingival MS.

## Data Availability Statement

The raw data supporting the conclusions of this article will be made available by the authors, without undue reservation.

## Ethics Statement

The studies involving human participants were reviewed and approved by the University of Pennsylvania Institutional Review Board. Written informed consent for participation was not required for this study in accordance with the national legislation and the institutional requirements. Written informed consent was not obtained from the individual(s) for the publication of any potentially identifiable images or data included in this article.

## Author Contributions

IP and JP conceived and designed the study. DL and JB collected and assembled the data. DL and JB wrote the manuscript. CW, JP, AP, and IP revised the manuscript and gave final approval. All authors contributed to the article and approved the submitted version.

## Funding

This research was funded by the Chair’s Radiation Oncology Summer Research Grant from the Hospital of the University of Pennsylvania.

## Conflict of Interest

AP has been a consultant for and received research funding from Astellas Pharma outside this study.

The remaining authors declare that the research was conducted in the absence of any commercial or financial relationships that could be construed as a potential conflict of interest.
