# Supplementary material for: Radiation Therapy for Chemotherapy Refractory Gingival Myeloid Sarcoma
Source: Front Oncol. 2021 May 10;11:671514. doi: 10.3389/fonc.2021.671514 (PMC8143974; doi:10.3389/fonc.2021.671514)
Supplement: Supplementary file 1 [file Table_1.docx]

Supplementary Table 1: Acute Myeloid Leukemia cytogenetic, molecular, and next generation sequencing data.

|  | **Patient 1** | **Patient 2** | **Patient 3** | **Patient 4** | | **Patient 5** | **Patient 6** |
| --- | --- | --- | --- | --- | --- | --- | --- |
| Cytogenetics at Diagnosis | Normal | Normal | Normal | Normal | Intermediate cytogenetics (46,XX,+1, del(1;22)(q10;q10) | | Isochrome 17q |
| Molecular/NGS data | NPM1/DNMT3a/TET2 pos,  FLT3 neg initially -> transformed positive | FLT3/NPM1 neg | FLT3 neg | FLT3 pos | | FLT3/LTM/NPM1 neg | FLT3 neg |

Abbreviations: NGS (next generation sequences)
